# Purpura Fulminans in the Setting of Klebsiella Pneumoniae Bacteremia and Acetaminophen Overdose

**DOI:** 10.7759/cureus.11633

**Published:** 2020-11-22

**Authors:** Vincent Nguyen, Janine A Myint, Marie Philipneri

**Affiliations:** 1 Department of Medicine, Division of Nephrology, Saint Louis University School of Medicine, Saint Louis, USA

**Keywords:** purpura fulminans, acute infectious purpura fulminans, disseminated intravascular coagulation, klebsiella pneumoniae, acetaminophen toxicity

## Abstract

Purpura fulminans (PF) is a rare, life-threatening disorder characterized by disseminated intravascular coagulation (DIC), circulatory collapse, and hemorrhagic cutaneous purpura. It typically occurs secondary to acute infections, usually meningococcal septicemia, although there are also congenital and acquired causes. We report a case of a 56-year old female who presented to our institution with clinical signs of PF in the setting of acetaminophen overdose and Klebsiella pneumoniae sepsis. Given the rarity of the disease, we also review cases of PF in similar clinical scenarios that have been described in the literature.

## Introduction

Purpura fulminans (PF) is a rare, life-threatening disorder with a mortality rate of up to 60% [1, 2]. It is characterized by disseminated intravascular coagulation (DIC), circulatory collapse, and dermal vascular thrombosis that results in hemorrhagic cutaneous purpura in the trunk and limbs [1, 3-6]. PF is typically seen in three clinical settings; inherited or acquired coagulation disorder, acute infection, or idiopathic [3, 7]. Most commonly, it is seen in children and is due to endotoxin-production from meningococcal disease [1, 3].

Cutaneous manifestations of PF include widespread ecchymoses, hemorrhagic bullae, and epidermal necrosis [6]. Patients initially present with erythema and petechiae that evolve into ecchymoses and purpuric plaques. With time, there is symmetric gangrene of distal extremities that extend proximally [4, 8]. Histopathology reveals dermal vascular thrombosis with the presence of microthrombi in the dermal blood vessels [1, 4, 8].

Because of the high mortality rate, it is imperative that timely diagnosis and treatment of PF are undertaken. It is also important to point out atypical presentations, such as PF in adults, and potential causes other than meningococcal disease or coagulation disorder. 

In our case report, we present a case of PF in the setting of Klebsiella pneumoniae and acetaminophen toxicity. 

## Case presentation

**Figure 1:**
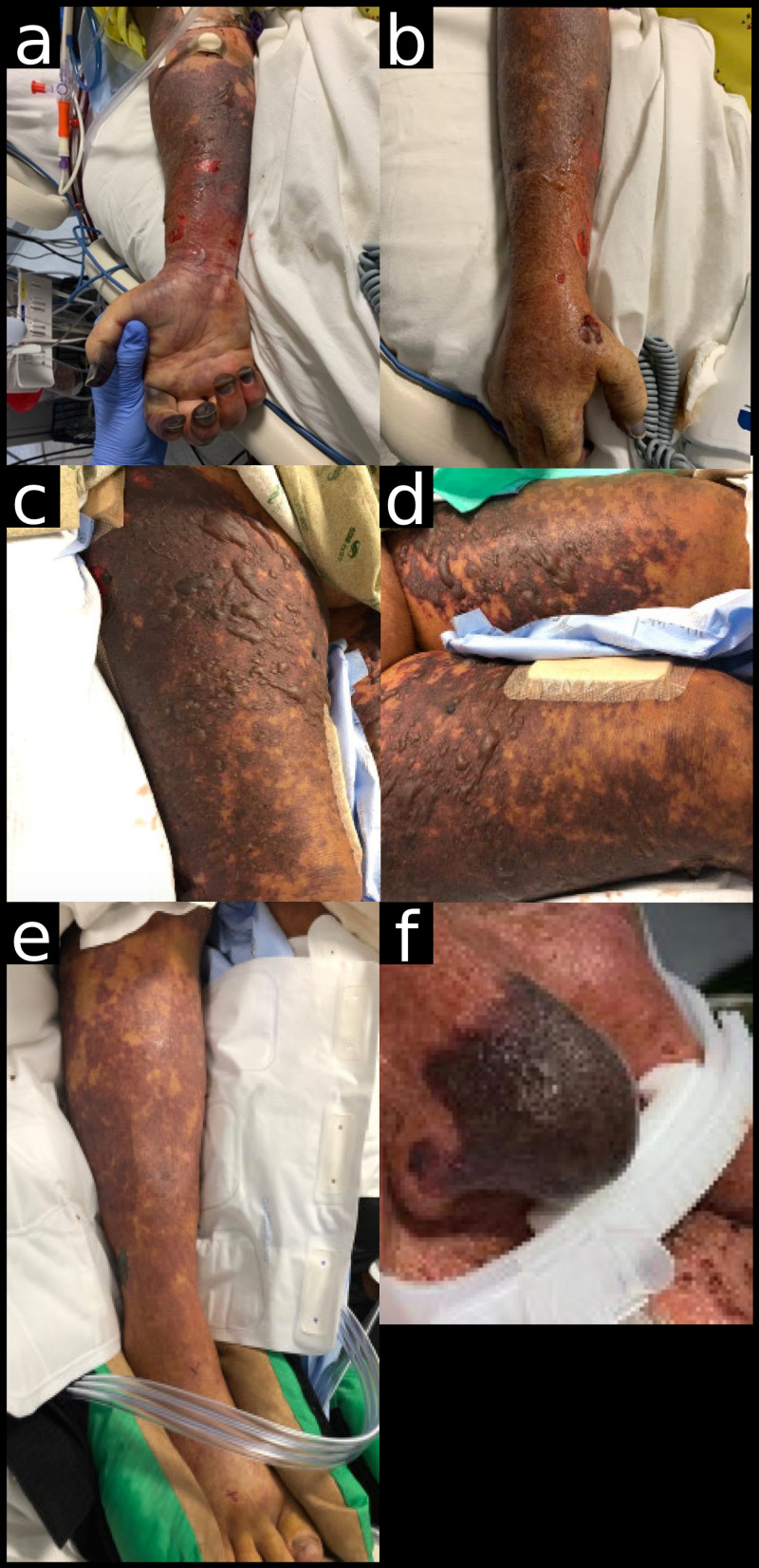
Early presentation of cutaneous findings. a,b. Retiform purpura on right arm with gangrenous changes in nails. c,d,e. Bullae on bilateral thighs and purpura extending down to toes. f. Purpura on the tip of the nose.

**Figure 2:**
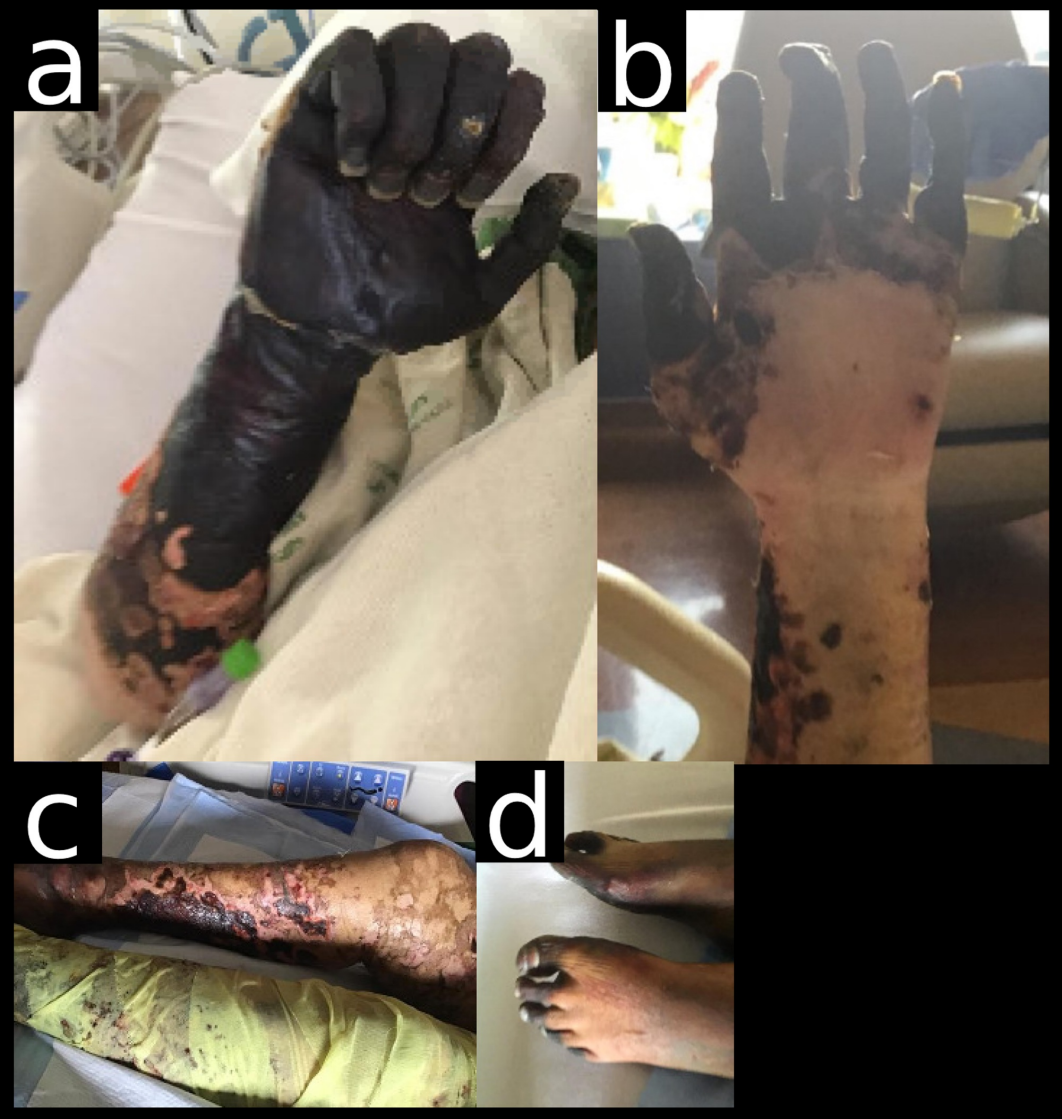
Late presentation of cutaneous findings. a. Dry gangrene of the right forearm/hand. b. Dry gangrene of the left hand. c. Gangrenous changes to the legs and distal toes bilaterally.

A 56-year-old female with a past medical history significant for depression, gastric cancer status post partial gastrectomy, and nonalcoholic steatohepatitis (NASH) presented as a transfer from an outside hospital for acute liver failure, concerning for acetaminophen overdose. She was noted to have altered mental status with laboratory studies revealing transaminitis and elevated acetaminophen level indicating “possible hepatic toxicity” per the Rumack-Matthew nomogram. She was admitted to the intensive care unit (ICU) with shock and multi-organ failure requiring vasopressors and mechanical ventilation. N-acetylcysteine (NAC) was administered for possible acetaminophen overdose, followed by Continuous Renal Replacement Therapy (CRRT) for worsening metabolic panel due to acute kidney injury. Physical examination was significant for cold bilateral distal extremities and skin mottling in the upper and lower extremities which progressed to involve the majority of the body over the course of several hours. Computerized tomography (CT) scans of chest, abdomen, and pelvis were notable for bilateral lower lobe lung consolidations with scattered ground-glass opacities, hepatomegaly, bowel wall thickening, and cortical necrosis in both kidneys. Blood cultures were positive for Klebsiella pneumoniae and she was started on meropenem and vancomycin. Laboratory tests revealed anemia, thrombocytopenia, elevated PT/PTT, elevated d-dimer, and low fibrinogen concerning for DIC. Skin mottling continued to worsen, later developing bullae over the bilateral anterior thighs. Dermatology was consulted and noted retiform purpura involving bilateral thighs, legs, feet, arms, hands, the tip of the nose, upper ear, and lower abdomen (Figure 1). Gangrenous changes were observed over the fingers and nails on both hands. Left upper arm biopsy demonstrated epidermal necrosis with extravasated red blood cells and scattered microthrombi in the papillary dermis. She was started on therapeutic heparin at this time although this was later discontinued. Plastic Surgery and Interventional Radiology were later consulted for management of gangrenous hands, but the decision was made to allow the necrotic areas to declare with no acute intervention (Figure 2). Thrombophilia and autoimmune evaluation were remarkable for positive lupus anticoagulant and decreased protein C and S activity, albeit she was receiving therapeutic heparin at this time. After initiation of antibiotics, she continued to improve and was stepped down from ICU to floor with wound care. Some return of blood flow to the proximal part of her hands was noted and she was discharged to a Long-term Acute Care (LTAC) facility. She was readmitted to the hospital less than a month later for right elbow disarticulation and left-hand amputation for progression of dry gangrene to wet gangrene. 

## Discussion

We describe a case of rapidly progressive retiform purpura in a patient who was found to have Klebsiella pneumoniae bacteremia. Two additional differential diagnoses were considered at the time of presentation; vasopressor-induced necrosis and vasculitis. Vasopressor-induced necrosis was a consideration given her gangrenous hands, although unlikely because of the hemorrhagic bullae formation in early presentation, rapid spread to her proximal extremities, and laboratory findings suggestive of DIC. Vasculitis was also considered but less likely given the unremarkable autoimmune workup. She did have a positive lupus anticoagulant, but according to Colling and Bendapudi, lupus anticoagulant can be positive at the time of presentation in patients with PF [1].

Several studies have noted that laboratory findings associated with PF include prolonged coagulation times, decreased fibrinogen, elevated d-dimer, abnormalities in protein C function, and thrombocytopenia [1, 5]. As for skin biopsy, the presence of microthrombi in dermal blood vessels is typical [1, 4, 5]. In our patient, DIC, decreased protein C and S activity, skin biopsy findings, and clinical improvement after antibiotic initiation made PF secondary to Klebsiella pneumoniae sepsis the most likely diagnosis. There have been a few case reports of PF in the setting of Klebsiella species infection (Table 1). In one case report of PF caused by Klebsiella pneumoniae, there was also involvement of the nose as seen in our patient, which was deemed an unusual location of PF manifestation. 

**Table 1:** Literature review of cases of PF from Klebsiella or Acetaminophen PF: Purpura fulminans

Author	Age at diagnosis	Likely Cause(s)	Management	Outcomes (including complications)
Tsubouchi et al. (2019) [9]	75-year old woman	Klebsiella Oxytoca	Intensive care	Death
Disse et al. (2018) [2]	17-day old neonate	Klebsiella Oxytoca sepsis from central venous catheter	Broad-spectrum antibiotics, ventilation, diuretics, protein C substitution, burn protocol	Limbs successfully preserved with scarring
Singh and Kampani (2018) [7]	19-year old female	Klebsiella Pneumoniae	IV fluids, broad-spectrum antibiotics, platelets	Unknown
Umar (2018) [10]	2-month old boy	Klebsiella Pneumoniae	Ceftriaxone, blood transfusions, FFP considered	Parents left against medical advice (AMA)
Guccione et al. (1993) [8]	32-year old woman	Acetaminophen and Alcohol toxicity	Heparin, Vitamin K, Cefuroxime	Resolution of Purpuric Lesions

This presentation is most consistent with acute infectious PF. Several researchers have noted that during infection, bacterial endotoxins induce coagulation secondary to consumption of protein C and S [5]. This results in an acquired hypercoagulable state that leads to thromboses in dermal vessels, DIC, and hemorrhagic skin necrosis. 

Our patient also had signs of concomitant acetaminophen toxicity that may have contributed to the development of PF. Although her transaminitis was not very high to suggest acute liver failure, the acetaminophen-induced liver injury may have led to further decline in protein C and S function. Guccione et al. published a report of PF with acquired protein C and S deficiency induced by alcohol and acetaminophen [8]. They speculated that the reduction in hepatic glutathione caused by acetaminophen toxicity led to the impairment of anticoagulation proteins synthesis, resulting in the activation of the DIC cascade and Purpura Fulminans [8].

In acute infectious PF, the treatment is to address the underlying cause with antibiotic therapy in addition to supportive care. Currently, there is no consensus on the treatment of PF. A few treatments that have been utilized include broad-spectrum antibiotics, anticoagulation, protein C, platelets, and FFP [1, 2, 5-8]. There may be a role for protein C replacement as seen in a small study where all patients survived despite high predicted mortality [11]. The RESOLVE study found that the use of protein C in the treatment of severe sepsis in children resulted in no significant improvement in organ failure or 28-day mortality but increased intracranial hemorrhage. Although this study did not analyze the use of protein C in PF specifically, there is enough overlap to suggest that protein C should be used with caution [5]. 

## Conclusions

Purpura fulminans is a rare, yet life-threatening condition that can result in DIC, hemodynamic collapse, and hemorrhagic cutaneous necrosis. PF is mostly seen in children in the setting of meningococcus bacteremia. Diagnostic delay can lead to major adverse clinical consequences to the patient, such as amputation of limbs and death. Our case presents a unique example of an adult with signs and symptoms of Purpura Fulminans in the setting of Klebsiella pneumoniae bacteremia and Acetaminophen toxicity. This case emphasizes the importance of considering Purpura fulminans in adults with acquired protein C and S deficiency states, including certain bacterial infections and compromised liver function.
